# Real-world data suggest effectiveness of the allogeneic mesenchymal stromal cells preparation MSC-FFM in ruxolitinib-refractory acute graft-versus-host disease

**DOI:** 10.1186/s12967-023-04731-1

**Published:** 2023-11-21

**Authors:** Halvard Bonig, Mareike Verbeek, Peter Herhaus, Krischan Braitsch, Gernot Beutel, Christoph Schmid, Nadine Müller, Gesine Bug, Michaela Döring, Arend von Stackelberg, Johanna Tischer, Francis Ayuk, Gerald Wulf, Udo Holtick, Lisa-Marie Pfeffermann, Bernd Jahrsdörfer, Hubert Schrezenmeier, Selim Kuci, Zyrafete Kuci, Anke Zens, Michael Tribanek, Robert Zeiser, Sabine Huenecke, Peter Bader

**Affiliations:** 1 Faculty of Medicine, Institute for Transfusion Medicine and Immunohematology, Goethe University, Frankfurt, Germany; 2 German Red Cross Blood Service BaWüHe, Institute Frankfurt, Frankfurt, Germany; 3 Department of Medicine, Division of Hematology, University of Washington, Seattle, WA USA; 4 School of Medicine, Technical University Munich, Klinikum Rechts Der Isar, Clinic and Policlinic for Internal Medicine III, Munich, Germany; 5 Hannover Medical School, Department of Hematology, and Stem Cell Transplantation, HemostasisHannover, Oncology Germany; 6 Augsburg University Hospital and Medical Faculty, Augsburg, Germany; 7 Universitätsklinikum Mannheim, Mannheim, Germany; 8 Department of Medicine 2, University Hospital, Goethe University, Frankfurt, Germany; 9 Universitätsklinik Für Kinder Und Jugendmedizin, Tübingen, Germany; 10 Charité, Universitätsmedizin Berlin, Berlin, Germany; 11 Department of Medicine III, LMU University Hospital, LMU Munich, Munich, Germany; 12 Klinik Für Stammzelltransplantation, Universitätsklinikum Hamburg-Eppendorf, Hamburg, Germany; 13 Hämatologie Und Medizinische Onkologie, Universitätsmedizin Göttingen, Göttingen, Germany; 14 Universitätsklinikum Köln, Cologne, Germany; 15 Institute for Clinical Transfusion Medicine and Immunogenetics, University of Ulm, Ulm, Germany; 16 Department of Pediatrics, Division for Stem Cell Transplantation and Immunology, Goethe University, Frankfurt, Germany; 17 Medac Gesellschaft Für Klinische Spezialpräparate mbH, Wedel, Germany; 18 Department Innere Medizin, Klinik Für Innere Medizin I, Universitätsklinikum Freiburg, Freiburg, Germany; 19 Department of Pediatrics, Division of Stem Cell Transplantation and Immunology, Goethe University Frankfurt, Theodor-Stern-Kai 7, 60590 Frankfurt am Main, Germany

**Keywords:** MSC-FFM, Mesenchymal stromal cells, Steroid-refractory, Ruxolitinib-refractory, Allogeneic haematopoietic stem cell transplantation

## Abstract

**Background:**

Patients with steroid-refractory acute graft-versus-host disease (aGvHD) not tolerating/responding to ruxolitinib (RR-aGvHD) have a dismal prognosis.

**Methods:**

We retrospectively assessed real-world outcomes of RR-aGvHD treated with the random-donor allogeneic MSC preparation MSC-FFM, available via Hospital Exemption in Germany. MSC-FFM is provided as frozen cell dispersion for administration as i.v. infusion immediately after thawing, at a recommended dose of 1–2 million MSCs/kg body weight in 4 once-weekly doses. 156 patients, 33 thereof children, received MSC-FFM; 5% had Grade II, 40% had Grade III, and 54% had Grade IV aGvHD. Median (range) number of prior therapies was 4 (1–10) in adults and 7 (2–11) in children.

**Results:**

The safety profile of MSC-FFM was consistent with previous reports for MSC therapies in general and MSC-FFM specifically. The overall response rate at Day 28 was 46% (95% confidence interval [CI] 36–55%) in adults and 64% (45–80%) in children; most responses were durable. Probability of overall survival at 6, 12 and 24 months was 47% (38–56%), 35% (27–44%) and 30% (22–39%) for adults, and 59% (40–74%), 42% (24–58%) and 35% (19–53%) for children, respectively (whole cohort: median OS 5.8 months).

**Conclusion:**

A recent real-world analysis of outcomes for 64 adult RR-aGvHD patients not treated with MSCs reports survival of 20%, 16% and 10% beyond 6, 12 and 24 months, respectively (median 28 days). Our data thus suggest effectiveness of MSC-FFM in RR-aGvHD.

**Supplementary Information:**

The online version contains supplementary material available at 10.1186/s12967-023-04731-1.

## Background

Acute graft-versus-host disease (aGvHD) remains the leading cause of treatment-related morbidity and mortality after allogeneic haematopoietic stem cell transplantation (HSCT) [1–3]. The incidence of Grade II–IV aGvHD has decreased over recent decades (from 40% in 1990–1995 to 28% in 2011–2015), in part due to improved donor selection and GvHD prophylaxis [4]. However, this is more than offset by the strong increase in the number of allogeneic HSCTs performed each year, with a more than tenfold increase in HSCT for malignant diseases observed in the European Society for Blood and Marrow Transplantation (EBMT) Registry from 1990 to 2015 [4].

High-dose corticosteroids are the established first-line treatment for patients with Grade II or higher aGvHD [2, 5, 6]. Approximately 30–60% of patients will respond, depending on aGvHD grade [7, 8]. Treatment options for patients with steroid-refractory aGvHD (SR-aGvHD) were limited for many years [2, 3, 5]. Conventional immunosuppressive drugs showed limited efficacy and a high rate of adverse events, with infections being common. Patients with SR-aGvHD typically succumbed to their underlying disease or to infectious complications quite rapidly, facilitated by the profound immunosuppression of GvHD medicines [1], with only about half of patients surviving to 6 months or beyond [2].

The Janus kinase (JAK) inhibitor ruxolitinib was recently authorized by the European Medical Association (EMA) and US Food and Drug Administration (FDA) for SR-aGvHD and has quickly become the new standard of care for this indication [9–11]. Objective overall response rates (ORR, i.e., complete or partial response) of 50% or greater have been reported across multiple studies, even in high-grade SR-aGvHD, with rates exceeding 80% in Grade II disease [12–14]. Patients who respond do so quite promptly (i.e., by Day 28); however, an early response was not correlated to survival. With regards to safety, ruxolitinib suppresses haematopoiesis and adaptive immune responses; thus, cytopenias are common treatment-emergent adverse events [13], as are reactivations of latent viral infections and other infectious complications [11, 12].

A proportion of patients do not respond to or do not tolerate ruxolitinib (herein referred to as ruxolitinib-refractory aGvHD [RR-aGvHD]). In the pivotal REACH2 trial, non-response was observed in 38% of patients at Day 28 and 60% by Day 56, and another 11% of patients had discontinued ruxolitinib by Day 28 due to adverse events [14]. Non-response was especially high in patients with Grade III or IV disease (44% and 47%, respectively). Real-world studies are less bleak, with ruxolitinib resistance or intolerance reported in 21% of adults [9] and 28–55% of children [15–17].

Outcomes for patients with RR-aGvHD are generally dismal. In the above-mentioned real-world study of adults with RR-aGvHD (N = 64), patients received a range of different and mostly off-label therapies including extracorporeal photopheresis, etanercept, mycophenolate mofetil or budesonide, given with or without concurrent ruxolitinib [9]. Of note, no patient in this series received mesenchymal stromal cell (MSC) therapy. Median survival was 28 days [21 days for ruxolitinib refractoriness, 50 days for ruxolitinib intolerance] [9]. Probability of overall survival (OS) was approximately 20%, 16% and 10% at 6, 12 and 24 months, respectively, despite administration of additional lines of immunosuppressive therapy in most patients [9]. These outcome data provide an approximate benchmark for initial assessment of the efficacy of novel therapeutic approaches to RR-aGvHD.

Allogeneic MSCs from bone marrow (BM) have been considered a promising approach to SR-aGvHD treatment ever since a case report was published by Le Blanc et al. in 2004 [18–20]. The basis for the use of MSCs in treating GvHD is their immunomodulatory effect [20, 21]. However, the development of MSC products with consistent pharmaceutical quality has been challenging, and large-scale randomized controlled studies demonstrating a positive risk–benefit balance are lacking [21–23].

We developed a BM-MSC product harvested from multiple donors (MSC-FFM), with stringent dose-to-dose equipotency and augmented immunomodulatory capacity compared with single-donor BM-MSC products [24]. Data indicating the clinical efficacy of this MSC product for the treatment of adult and paediatric patients with SR-aGvHD have been reported previously [25, 26]. In brief, for 92 children and adults with aGvHD that was refractory to steroids (and often additional therapies) receiving this MSC therapy, ORR was 82% and 81% at first and last follow-up, respectively. Six-month OS was 64%. Safety, both acute and long-term, appeared to be good [25, 26].

Other MSC products, predominantly derived from bone marrow (BM), sometimes also from adipose tissue, have been studied for SR-aGvHD with varying success [21, 22]. In a recent Phase III trial, remestemcel-L (Ryoncil®) failed to achieve significantly greater durable complete response or overall response versus placebo in patients with SR-aGvHD (N = 260) [27]. However, it was more efficacious than placebo in subgroups with high-grade aGvHD, liver involvement or children/adolescents [27]. In a real-world study of Temcell® (licensed for GvHD treatment in Japan), a subgroup of 151 evaluable patients received Temcell^®^ for SR-aGvHD [28]. Of these patients, 61% achieved an overall response on Day 28, which was predictive of survival. Decidua-derived MSCs have recently been proposed as an alternative to BM-MSCs with potentially even higher pharmacological activity [29], but unlike MSC-FFM have thus far not been developed to pharmaceutical scale. For a recent review of smaller trials of MSCs for aGvHD treatment, we direct the reader to a Cochrane review by Fisher et al. [21].

A Phase III, prospective, randomized controlled trial (IDUNN) is underway to assess first-line treatment with MSC-FFM versus best available therapy (BAT), including ruxolitinib, in adults and adolescents with SR-aGvHD after allogeneic HSCT (NCT04629833). Enrolling patients in more than 40 transplant centres across 5 European countries, the trial will test for superiority of MSC-FFM versus BAT with regard to ORR at Day 28. A similarly designed phase III trial for children was just approved but is not yet enrolling (BALDER, NCT06075706).

Currently, MSC-FFM is available for treatment of patients with SR-aGvHD in Germany under Hospital Exemption. This MSC preparation has also been used on a named-patient basis in other European countries. Since the approval of ruxolitinib, use of MSC-FFM has been almost exclusively restricted to patients with RR-aGvHD.

We report here a retrospective analysis of outcomes for 156 heavily pre-treated patients with RR-aGvHD who received MSC-FFM as salvage therapy under Hospital Exemption in Germany or on a named-patient basis outside Germany.

## Methods

### Data collection

This was a multi-site retrospective analysis. Pseudonymized data are actively collected by the licence holder of the Hospital Exemption for all patients receiving MSC-FFM in support of the regulator-required periodic product safety update reporting (PSUR). Since January 2018, the licence holder has established a Data Surveillance Programme aiming to collect efficacy and safety data of these patients; these data are reported annually to the Paul-Ehrlich-Institute as a requirement to maintain the Hospital Exemption for this MSC product.

Patients (or legal representatives) consented to treatment with MSC-FFM; the required collection and analysis of pseudonymized outcome data by the pharmaceutical manufacturer does not require permission from an ethics committee. Data were documented by treating physicians and, where needed, flying study nurses (who provide project support across multiple sites) in an electronic case report form.

From this database, efficacy and safety results were extracted for patients with SR-aGvHD who had been documented to have received ruxolitinib during the course of their GvHD management prior to MSC administration. These patients were considered to have RR-aGvHD because MSC-FFM was ordered by the treating physician as salvage therapy.

Patients were followed-up for 24 months from the first administration of MSC-FFM.

### Treatment

In brief, MSC-FFM (medac Gesellschaft für klinische Spezialpräparate mbH) is manufactured from pooled BM mononuclear cells from eight HLA-disparate healthy donors. MSCs are selected by plastic adherence, expanded in platelet lysate–enriched media in 2D culture to the end of passage 3, then frozen in saline-albumin with DMSO at a final concentration of 10% v/v until immediately prior to infusion [24].

MSC-FFM was dosed at 1–2 million MSCs/kg bodyweight as a once-weekly intravenous infusion for four doses. Further doses were ordered in a minority of patients on a named-patient basis in order to further improve or sustain response.

### Outcomes

Acute GvHD staging and grading followed international conventions (e.g., the Mount Sinai Acute GvHD International Consortium [MAGIC] criteria) [30]. Similarly, grading of quality of response followed established criteria. Complete response was defined as complete resolution of signs, symptoms and laboratory evidence of aGvHD, albeit not necessarily cessation of concomitant GvHD medication. Partial response was defined as improvement by at least one stage in at least one organ system without progression in another. Very good partial response was defined as improvement in at least one organ by at least one stage without progression in any other organ and absence of all aGvHD symptoms except Stage 1 disease in any organ.

### Data analysis

The number and percentage of patients with overall response per visit is given together with the exact 95% Clopper-Pearson confidence interval (CI).

OS was defined as the length of time between start of MSC-FFM treatment and the date of death due to any cause within the 24-month study period. Patients alive at their last follow-up were censored. OS rates and associated 95% CIs were calculated by applying Kaplan–Meier methods.

The median follow-up in months for patients who survived was calculated by Kaplan–Meier methods based on OS, treating the date of death as the censoring date and end of follow-up as an event.

## Results

### Patient characteristics

Between December 2017 and February 2023, 156 patients, including 33 children and adolescents (< 18 years of age), received MSC-FFM for RR-aGvHD. Thirty-two German sites contributed 139 patients; the remaining 17 patients came from seven transplant centres in France, Hungary, Norway, Sweden and Switzerland. A full list of participating centres and treating physicians is given in Additional file 1: Table S1.

The adult cohort was 41% female. Age ranged from 19 to 79 years (median 55 years; interquartile range [IQR] 45–63 years). Except for one patient, all had been diagnosed with malignant disease (three patients had missing information); of these, 76% (n = 90) received HSCT for acute myeloid leukaemia (AML), advanced myelodysplastic syndrome (MDS), or myeloproliferative neoplasm (MPN) and 13% (n = 15) for acute lymphoblastic leukaemia (ALL).

In the paediatric cohort, 49% of patients were female. All age groups were represented, with a range from 0 to 17 years (median 9 years; IQR 6–12 years). Overall, 58% of paediatric patients (n = 19) had malignant disease as the indication for allogeneic HSCT. Of these, 42% (n = 8) had ALL and 32% (n = 6) had AML. The remaining 42% of patients (n = 14) had a non-malignant disease as their indication for HSCT. Table 1 shows baseline demographic and underlying disease data in more detail.

**Table 1:** Demographic data and disease characteristics by age group

Patients	Children and adolescents (< 18 years)	Adults (≥ 18 years)	Total cohort
	n = 33	n = 123	N = 156
Sex, n (%)			
Female	16 (48.5%)	50 (40.7%)	66 (42.3%)
Male	17 (51.5%)	73 (59.3%)	90 (57.7%)
Age, years			
Mean (SD)	8.9 (4.9)	52.5 (14.2)	43.3 (22.0)
Median (Q1, Q3)	9.0 (6.0, 12.0)	55.0 (45.0, 63.0)	49.5 (22.5, 62.0)
Min, max	0, 17	19, 79	0, 79
ICH age group, n (%)			
Infants/toddlers (28 days to 23 months)	2 (6.1%)	NA	2 (1.3%)
Children (2 to 11 years)	18 (54.5%)	NA	18 (11.5%)
Adolescents (12 to 17 years)	13 (39.4%)	NA	13 (8.3%)
Adults (18 to 64 years)	NA	99 (80.5%)	99 (63.5%)
Elderly people; 65 to 74 years	NA	22 (17.9%)	22 (14.1%)
Elderly people; 75 to 84 years	NA	2 (1.6%)	2 (1.3%)
Body weight, kg			
Patients with data	32	122	154
Mean (SD)	32.7 (18.0)	68.6 (14.6)	61.1 (21.2)
Median (Q1, Q3)	27.5 (19.0, 44.0)	69.0 (56.0, 78.0)	64.0 (50.0, 75.0)
Missing	1	1	2
Primary diagnosis, n (%)			
Malignant haematopoietic	19 (57.6%)	119 (96.7%)	138 (88.5%)
Acute leukaemia of ambiguous lineage	0 (0.0%)	2 (1.6%)	2 (1.3%)
AML or related neoplasm	6 (18.2%)	43 (35.0%)	49 (31.4%)
ALL	8 (24.2%)	15 (12.2%)	23 (14.7%)
AMML	0 (0.0%)	1 (0.8%)	1 (0.6%)
NHL	2 (6.1%)	11 (8.9%)	13 (8.3%)
MDS/MPN	3 (9.0%)	47 (38.2%)	50 (32.1%)
Non-malignant (NOS)	14 (42.4%)	1 (0.8%)	15 (9.6%)
Missing	0 (0.0%)	3 (2.4%)	3 (1.9%)

AML, acute myeloid leukaemia; AMML, acute myelomonocytic leukaemia; ALL, acute lymphocytic leukaemia; ICH, International Council for Harmonisation; MDS, myelodysplastic syndrome; MPN, myeloproliferative neoplasm; NHL, non-Hodgkin lymphoma; NOS, not otherwise specified; NA, not applicable, SD, standard deviation

All patients were severely affected by aGvHD and all were steroid-refractory (SR-aGvHD) as well as ruxolitinib-refractory. Of the adult patients, six (5%) had Grade II aGvHD, 52 (42%) had Grade III, and 63 (51%) had Grade IV, and in two patients (1.6%) no grading was reported. In the paediatric subgroup, 2 (6%) had Grade II, 10 (30%) had Grade III, and 21 (64%) had Grade IV aGvHD. At the time of treatment with MSC-FFM, patients had been heavily pre-treated: adults had received and failed to adequately respond to a median of three (range 0–9) and children six (range 1–10) prior lines of SR-aGvHD treatment besides ruxolitinib (see Table 2).

**Table 2:** Details of aGvHD grade and treatment prior to MSC-FFM therapy by age group

	Children < 18 years (n = 33)		Adults ≥ 18 years (n = 123)	Overall (N = 156)
aGvHD Grade, n (%)				
II	2 (6.1%)		6 (4.9%)	8 (5.1%)
III	10 (30.3%)		52 (42.3%)	62 (39.7%)
IV	21 (63.6%)		63 (51.2%)	84 (53.8%)
Missing	0 (0.0%)		2 (1.6%)	2 (1.3%)
Prior aGvHD therapies, n (%) Number				
1	0 (0.0%)		1 (0.8%)	1 (0.6%)
> 1	33 (100.0%)		122 (99.2%)	155 (99.4%)
Median	7.0		4.0	4.0
Min, max	2, 11		1, 10	1, 11
Time from last HSCT to first MSC-FFM administration, days				
n	32		123	155
Mean (SD)	105.7 (80.4)		141.6 (110.4)	134.2 (105.7)
Median (Q1, Q3)	83.0 (50.0, 137.0)		112.0 (69.0, 181.0)	106.0 (64.0, 174.0)
Min, Max	30, 346		17, 671	17, 671
Missing	1		0	1

aGvHD, acute graft-versus-host disease; HSCT, haematopoietic stem cell transplantation, SD, standard deviation

No clear standard was predefined for the sequence of therapies applied for SR-aGvHD: all agents and combinations were allowed. Per the study design, enrolled patients had received ruxolitinib at some point as part of their SR-aGvHD treatment prior to MSC-FFM infusion. In accordance with dosing recommendations given in the product information for MSC-FFM (1–2 million cells/kg body weight, once weekly for 4 weeks), the median dose was 1.18 million cells/kg body weight, the median number of doses was four, and the median inter-dose interval was 7 days.

### Safety

The tolerability of MSC-FFM was good: only five adverse drug reactions were reported in three adult patients. One case of chills and one case of BK virus cystitis were reported. In a third patient, MSC infusion was associated with an increase in C-reactive protein (two instances) and nausea. None of these reactions led to cessation of MSC-FFM or dose reductions.

### Response

Overall response rate at Day 28 was 49% (95% CI 41–58%) in the whole cohort, 46% (95% CI 36–55%) in adults, and 64% (95% CI 45–80%) in children (Table 3).

**Table 3:** Response over time

All patients, n [%], (95% CI^a^)	Day 28	Day 60	Day 180
	N = 156	N = 155	N = 147
Overall response^b^	77 [49.4%] (41.3%, 57.5%)	76 [49.0%] (40.9%, 57.2%)	58 [39.5%] (31.5%, 47.8%)
Complete response	21 [13.5%] (8.5%, 19.8%)	41 [26.5%] (19.7%, 34.1%)	45 [30.6%] (23.3%, 38.7%)
Partial response	56 [35.9%] (28.4%, 44.0%)	35 [22.6%] (16.3%, 30.0%)	13 [8.8%] (4.8%, 14.6%)
Very good partial response	22 [14.1%] (9.1%, 20.6%)	12 [7.7%] (4.1%, 13.1%)	5 [3.4%] (1.1%, 7.8%)
Other partial response	34 [21.8%] (15.6%, 29.1%)	23 [14.8%] (9.6%, 21.4%)	8 [5.4%] (2.4%, 10.4%)
No response	79 [50.6%] (42.5%, 58.7%)	79 [51.0%] (42.8%, 59.1%)	89 [60.5%] (52.2%, 68.5%)

Children/adolescents, n[%], (95% CI^a^)	Day 28	Day 60	Day 180
	n = 33	n = 33	n = 30
Overall response^b^	21 [63.6%] (45.1%, 79.6%)	17 [51.5%] (33.5%, 69.2%)	14 [46.7%] (28.3%, 65.7%)
Complete response	3 [9.1%] (1.9%, 24.3%)	11 [33.3%] (18.0%, 51.8%)	11 [36.7%] (19.9%, 56.1%)
Partial response	18 [54.5%] (36.4%, 71.9%)	6 [18.2%] (7.0%, 35.5%)	3 [10.0%] (2.1%, 26.5%)
Very good partial response	5 [15.2%] (5.1%, 31.9%)	3 [9.1%] (1.9%, 24.3%)	1 [3.3%] (0.1%, 17.2%)
Other partial response	13 [39.4%] (22.9%, 57.9%)	3 [9.1%] (1.9%, 24.3%)	2 [6.7%] (0.8%, 22.1%)
No response	12 [36.4%] (20.4%, 54.9%)	16 [48.5%] (30.8%, 66.5%)	16 [53.3%] (34.3%, 71.7%)

Adults, n[%], (95% CI^a^)	Day 28	Day 60	Day 180
	n = 123	n = 122	n = 117
Overall response^b^	56 [45.5%] (36.5%, 54.8%)	59 [48.4%] (39.2%, 57.6%)	44 [37.6%] (28.8%, 47.0%)
Complete response	18 [14.6%] (8.9%, 22.1%)	30 [24.6%] (17.2%, 33.2%)	34 [29.1%] (21.0%, 38.2%)
Partial response	38 [30.9%] (22.9%, 39.9%)	29 [23.8%] (16.5%, 32.3%)	10 [8.5%] (4.2%, 15.2%)
Very good partial response	17 [13.8%] (8.3%, 21.2%)	9 [7.4%] (3.4%, 13.5%)	4 [3.4%] (0.9%, 8.5%)
Other partial response	21 [17.1%] (10.9%, 24.9%)	20 [16.4%] (10.3%, 24.2%)	6 [5.1%] (1.9%, 10.8%)
No response	67 [54.5%] (45.2%, 63.5%)	63 [51.6%] (42.4%, 60.8%)	73 [62.4%] (53.0%, 71.2%)

^a^95% confidence intervals (CIs) show the exact (Clopper-Pearson) confidence limits for the binomial proportion

^b^Overall response includes complete response, very good partial response, and other partial response

Most responses were durable, resulting in ORR of 49% (95% CI 41–57%) in the whole cohort; 48% (95% CI 39–58%) in adults, and 52% (33–69%) for children at Day 60, and of 40% (95% CI 32–48%) in the whole cohort, 38% (95% CI 29–47%) for adults and 47% (95% CI 28–66%) for children at Day 180.

While most responses were partial on Day 28, the depth of the response improved over time: 78% (45/58) of all responses were a complete response at Day 180. Responses over time indicate that aGvHD improved in both adults and children with MSC-FFM treatment (Table 3).

Response rate and depth of response did not differ by dose, i.e., within the narrow dose range applied in this case series, no dose effect was observed (data not shown).

### Survival

**Fig. 1:**
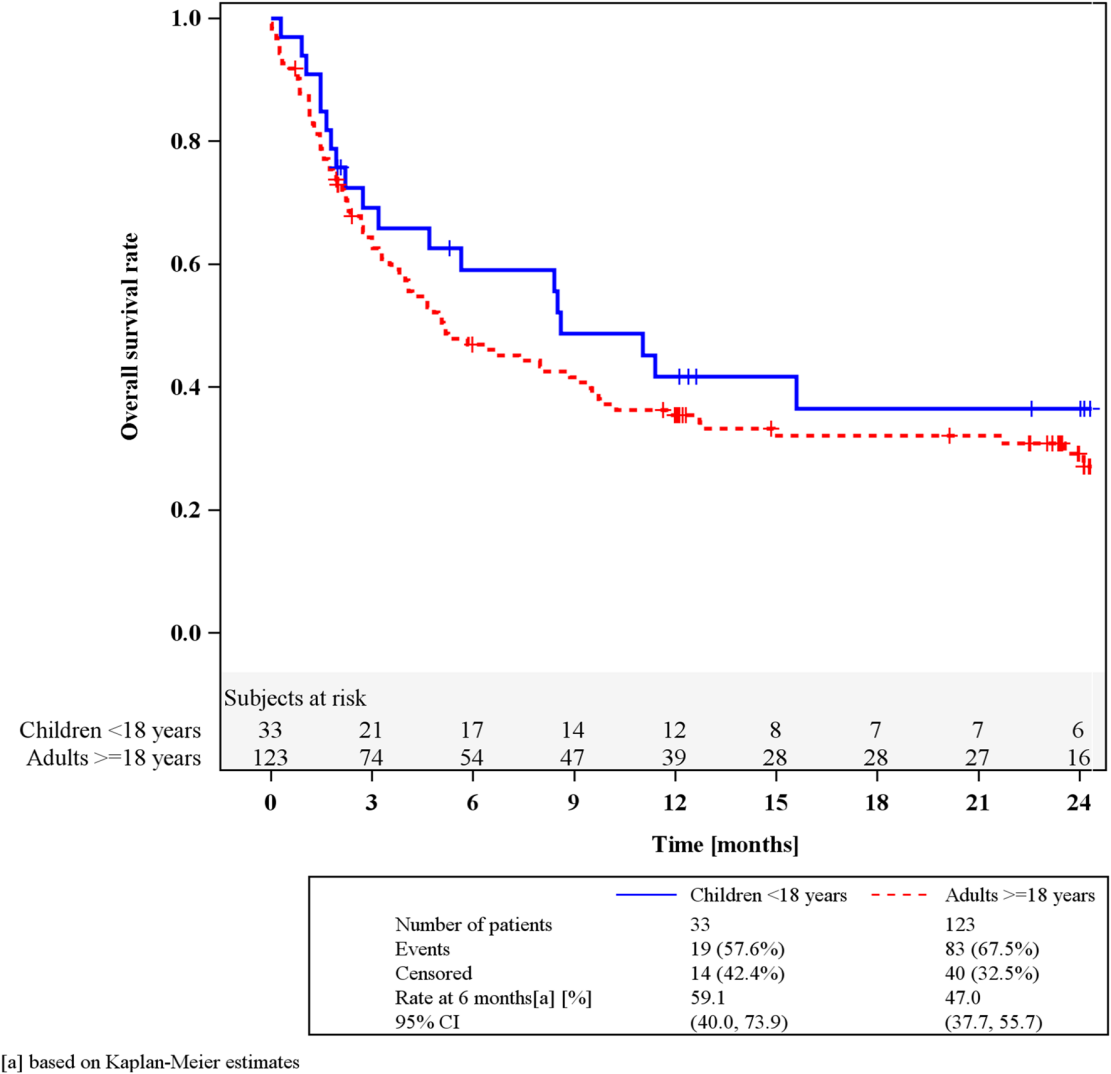
Kaplan–Meier curves for overall survival in patients with RR-aGvHD. Curves indicate overall survival (OS) for children (blue) and adults (red) with ruxolitinib-refractory acute graft-versus-host disease treated with MSC-FFM

OS at 6, 12 and 24 months was 47% (95% CI 38–56%), 35% (95% CI 27–44%) and 30% (95% CI 22–39%) for adults, and 59% (95% CI 40–74%), 42% (95% CI 24–58%) and 35% (19–53%) for children, respectively (Fig. 1 and Table 4). This indicates that the better ORR of children than adults was clinically meaningful also in terms of survival.

**Table 4:** Kaplan–Meier estimates of overall survival by age group

	No. at risk	Cumulative events/censored	Survival rate, %	(95% CI)
Children < 18 years (n = 33)				
At 0 months	33	0/0	100.0	(100.0, 100.0)
At 3 months	21	10/2	69.2	(50.2, 82.1)
At 6 months	17	13/3	59.1	(40.0, 73.9)
At 9 months	14	16/3	48.7	(30.2, 64.8)
At 12 months	12	18/3	41.7	(24.2, 58.4)
At 24 months	6	20/8	35.4	(18.6, 52.7)
Adults ≥ 18 years (n = 123)				
At 0 months	123	1/0	99.2	(94.4, 99.9)
At 3 months	74	44/6	63.5	(54.2, 71.4)
At 6 months	54	63/7	47.0	(37.7, 55.7)
At 9 months	47	69/7	41.6	(32.6, 50.4)
At 12 months	39	76/10	35.4	(26.8, 44.1)
At 24 months	17	84/30	30.3	(22.0, 39.1)

CI, confidence interval

Median OS across the whole cohort was 5.8 months. Day 28 response rates were highly predictive of survival at 6, 12 and 24 months.

A number of caveats notwithstanding, which are discussed below and which limit the strength of this analysis, we benchmarked survival rates for RR-aGvHD patients treated with MSC-FFM against published real-world OS estimates for adult RR-aGvHD patients receiving BAT after ruxolitinib failure: OS was 20% at 6 months, 16% at 12 months, and 10% at 24 months [9], much lower than in our cohort. Similarly, in that historical cohort, median survival was 28 days, compared to 5.8 months observed in our cohort.

### Deaths

Overall, 102 patients (65%) died over the course of the observation, with a median follow-up of surviving patients of 26.7 months. Of the patients who were classified as non-responders at day 28, 81% (64/79) died during the reporting period compared with 49% (38/77) of those who improved after receiving MSC-FFM. Transplantation-related mortality—predominantly due to GvHD—was the leading course of death (n = 78). Only 11 deaths were related to the primary underlying disease.

## Discussion

While ruxolitinib has meaningfully improved outcomes for patients with SR-aGvHD, those patients who fail to respond to or are intolerant of ruxolitinib typically have a dismal prognosis. Given the poor outcomes recently reported for patients with RR-aGvHD receiving BAT [9], new treatment options to treat aGvHD safely and effectively in this patient population are urgently needed.

In our cohort of heavily pre-treated patients whose SR-aGvHD could not be controlled with ruxolitinib, treatment with the unique MSC preparation MSC-FFM in a later line of therapy was associated with remarkable clinical outcomes. Clinical ORR at Day 28 was 49% for the whole cohort, with 46% of adults and 64% of children responding to treatment. This is meaningfully better than a historical external reference cohort of adult RR-aGvHD patients receiving BAT (excluding MSCs) reported by Abedin et al. In that cohort, patients who could receive additional treatment had a probability of response of 36%, which translated into a survival probability for the whole cohort of only 10% and a median OS of only 28 days [9]. While we would argue that the data thus seem to suggest efficacy of MSC-FFM in RR-aGvHD, we cannot exclude that certain biases have affected the outcome. Specifically, the treating physicians might have inadvertently selected their “best” patients for MSC-FFM treatment. Secondly, during the lag time between the establishment of ruxolitinib refractoriness/intolerance and the decision to pursue treatment with MSCs some of those patients with the highest disease burden might already have died, leaving a relatively lower-risk population which could even receive MSC-FFM. These and certain other differences between the cohorts reported by Abedin et al. [9] and ourselves thus limit the direct comparison of the outcome data.

While the goal of this manuscript is not to provide a scientific discussion of MSCs in aGvHD per se, but to report outcomes with a specific proprietary MSC preparation of stringent GMP quality, the possibility to source MSCs from tissues other than BM, especially decidua-derived MSCs, which potentially even might be more potent in refractory GvHD than the BM-derived MSC preparation discussed here, should be acknowledged [29], as well as MSCs of different provenance may have different safety/adverse effect profiles [31]. That said, MSC-FFM is distinguished not only by young mitotic age, high efficacy and stringent batch-to-batch equipotency, but by the expansive pharmaceutical development package supporting its quality, and its regulatory status. Thus MSC-FFM is a market-ready off-the-shelf medicinal product. Success of the currently ongoing phase III trials in adults (IDUNN, NCT04629833) and children (BALDER, NCT06075706) provided, a marketing authorization in the EMA region will be sought. Conflicting evidence as effects of freeze-thawing of (BM-)MSCs for their immunomodulatory potential has been provided [32, 33]; the decision to deliver MSC-FFM as a frozen product for immediate infusion after thawing was guided by clinical and logistical feasibility [34] after potent immunomodulation of freshly thawed MSC-FFM MSCs was consistently documented.

Our study has several additional limitations that are characteristic for retrospective, real-world analyses, very specifically lack of defined in- and exclusion criteria and lack of granularity of accessible clinical data. For instance, we could not determine which GvHD medications were given concomitantly with or beyond administration of MSC therapy, nor assess steroid weaning. Accordingly, the observed clinical improvement that we refer to as ‘response’ to MSC-FFM might only partly be attributable to treatment with MSCs. The clinical improvement observed could, in theory, be completely or partly a late response to earlier lines of therapy or even spontaneous improvement. However, we do not consider this very likely because the pivotal trials of ruxolitinib recorded very few delayed responses [13, 14]. For all these reasons, this retrospective study may well overestimate the efficacy of MSC-FFM in RR-aGvHD.

With respect to the real-world study by Abedin et al., it should also be noted that the majority of their patients had not received ruxolitinib as first-line treatment after establishment of steroid refractoriness, thus their cohort may not represent the maximum possible benefit that can be derived from ruxolitinib [9]. However, the same may be said for the cohort described by us: with few exceptions, additional lines of treatment had been administered before and after ruxolitinib. At the time of study by Abedin et al., letermovir would not have been available for the majority of patients. Hence, more deaths from cytomegalovirus would be expected, possibly contributing to the extremely poor outcomes for RR-aGvHD patients in that series.

A limitation applicable to both Abedin’s [9] and our studies relates to the respective definitions of RR-aGvHD used (the previous use of ruxolitinib and a physician order of MSC-FFM as salvage therapy). A stringent definition of RR-aGvHD has recently been proposed by an international expert panel to aid the identification of patients who do not respond to ruxolitinib for use in future trials of later lines of therapy [35].

A prospective and ideally randomized and controlled trial is required to formally investigate the potential benefits and risks of MSC-FFM in RR-aGvHD. The ability to taper steroids, an important endpoint from the patients’ perspective, could be part of the study design.

The higher response rates, deeper responses and greater OS probability of children compared with adults in our cohort are in accordance with the previously reported greater responsiveness of paediatric aGvHD to MSC therapy [23, 26]. Day 28 response rates were highly predictive of survival, meaning that responses often translated into patient-relevant outcomes. Within the narrow dose range provided, no dose effect was observed with regard to response rate or depth.

In leukaemia patients, treatment with MSC-FFM appears not to interfere with the graft-versus-leukaemia effect of HSCT, since relapse of the malignant disease was infrequent. Also otherwise, MSC-FFM showed an excellent safety profile. No new safety signals were detected in this heavily pre-treated adult and paediatric patient population with mostly high-grade RR-aGvHD.

## Conclusions

In summary, we report that administration of the random-donor off-the-shelf MSC preparation MSC-FFM, generated from multiple donors using a proprietary pooling approach, led to very promising response and survival outcomes in a cohort of 156 heavily pre-treated patients with high-grade RR-aGvHD. Overall, outcomes markedly exceeded expectations based on historical data from patients with RR-aGvHD receiving other GvHD treatment modalities, and the risk-to-benefit ratio of MSC-FFM was extremely favourable. Formal clinical trials are needed to confirm the potential benefit of MSC-FFM treatment for aGvHD in this patient group for whom approved treatment options are limited. Meanwhile, a controlled trial of MSC-FFM vs. best available treatment in adults with severe steroid-refractory aGvHD (NCT04629833), with Ruxolitinib as the most frequently selected treatment in the control arm, are ongoing and will establish its role in earlier stages of aGvHD.

### Supplementary Information

**Additional file 1.** Participating centers.

## Data Availability

All data generated or analysed during this study are included in this published article and its supplementary information files.

## Abbreviations

aGvHD: Acute graft-vs.-host disease
AML: Acute myeloid leukemia
ALL: Acute lymphoblastic leukemia
BAT: Best available treatment
BK: (Betapolyomavirus first isolated from a patient with the initials B.K.)
CI: Confidence interval
DMSO: Dimethylsulfoxide
EBMT: European Society for Blood and Marrow Transplantation
EMA: European medicines agency
FDA: US Food and Drug Administration
HSCT: Hematopoietic stem cell transplantation
IQR: Interquartile range
JAK: Janus kinase
MAGIC: Mount Sinai Acute GvHD International Consortium
MDS: Myelodysplastic syndrome
MPN: Myeloproliferative neoplasm
MSC: Mesenchymal stromal cells
MSC-FFM: MSC Frankfurt on Main; tentative name of the proprietary MSC preparation used in this study
ORR: Overall response rate
OS: Overall survival
PSUR: Periodic product safety update reporting
RR-aGvHD: Ruxolitinib refractory aGvHD
SR-aGvHD: Steroid resistant aGvHD
