# Correction: Real‑world data suggest effectiveness of the allogeneic mesenchymal stromal cells preparation MSC‑FFM in ruxolitinib‑refractory acute graft‑versus‑host disease

**DOI:** 10.1186/s12967-024-05348-8

**Published:** 2024-06-13

**Authors:** Halvard Bonig, Mareike Verbeek, Peter Herhaus, Krischan Braitsch, Gernot Beutel, Christoph Schmid, Nadine Müller, Gesine Bug, Michaela Döring, Arend von Stackelberg, Johanna Tischer, Francis Ayuk, Gerald Wulf, Udo Holtick, Lisa‑Marie Pfeffermann, Bernd Jahrsdörfer, Hubert Schrezenmeier, Selim Kuci, Zyrafete Kuci, Anke Zens, Michael Tribanek, Robert Zeiser, Sabine Huenecke, Peter Bader

**Affiliations:** 1 Faculty of Medicine, Institute for Transfusion Medicine and Immunohematology, Goethe University, Frankfurt, Germany; 2 German Red Cross Blood Service BaWüHe, Institute Frankfurt, Frankfurt, Germany; 3 Department of Medicine, Division of Hematology, University of Washington, Seattle, WA USA; 4 School of Medicine, Technical University Munich, Klinikum Rechts Der Isar, Clinic and Policlinic for Internal Medicine III, Munich, Germany; 5 Department of Hematology, and Stem Cell Transplantation, Hemostasis, Oncology, Hannover Medical School, Hannover, Germany; 6 Augsburg University Hospital and Medical Faculty, Augsburg, Germany; 7 Universitätsklinikum Mannheim, Mannheim, Germany; 8 Department of Medicine 2, University Hospital, Goethe University, Frankfurt, Germany; 9 Universitätsklinik Für Kinder Und Jugendmedizin, Tübingen, Germany; 10 Charité, Universitätsmedizin Berlin, Berlin, Germany; 11 Department of Medicine III, LMU University Hospital, LMU Munich, Munich, Germany; 12 Klinik Für Stammzelltransplantation, Universitätsklinikum, Hamburg, Germany; 13 Hämatologie Und Medizinische Onkologie, Universitätsmedizin Göttingen, Göttingen, Germany; 14 Universitätsklinikum Köln, Cologne, Germany; 15 Institute for Clinical Transfusion Medicine and Immunogenetics, University of Ulm, Ulm, Germany; 16 Department of Pediatrics, Division for Stem Cell Transplantation and Immunology, Goethe University, Frankfurt, Germany; 17 Medac Gesellschaft Für Klinische Spezialpräparate mbH, Wedel, Germany; 18 Department Innere Medizin, Klinik Für Innere Medizin I, Universitätsklinikum Freiburg, Freiburg, Germany; 19 Department of Pediatrics, Division of Stem Cell Transplantation and Immunology, Goethe University Frankfurt, Theodor-Stern-Kai 7, 60590 Frankfurt am Main, Germany

**Correction: Journal of Translational Medicine (2023) 21:837** 10.1186/s12967-023-04731-1

Following publication of the original article [1], we have been notified that the author’s last name was published incorrectly.

It is now:

Krischan Braitssch

It should be:

Krischan Braitsch
