# Supplementary material for: Real-world data suggest effectiveness of the allogeneic mesenchymal stromal cells preparation MSC-FFM in ruxolitinib-refractory acute graft-versus-host disease
Source: J Transl Med. 2023 Nov 21;21:837. doi: 10.1186/s12967-023-04731-1 (PMC10664468; doi:10.1186/s12967-023-04731-1)
Supplement: Supplementary file 1 [file 12967_2023_4731_MOESM1_ESM.docx]

No	Center	Country	Physician	No of Patients
0102	Klinikum rechts der Isar der Technischen Universität München / München	Germany	Dr. Mareike Verbeek	33
0103	Medizinische Hochschule Hannover / Hannover	Germany	Dr. Gernot Beutel;	15
0113	Klinikum Augsburg / Augsburg	Germany	Prof. Dr. Christoph Schmid	9
0109	Universitätsmedizin Mannheim / Mannheim	Germany	Dr. Nadine Müller	8
0101	Universitätsklinikum Frankfurt / Frankfurt	Germany	Prof. Bader; Dr. Gesine Bug;	7
0108	Universitätsklinik für Kinder- und Jugendmedizin / Tübingen	Germany	Dr. Michaela Döring	7
0112	Charité – Universitätsmedizin Berlin / Berlin	Germany	PD Dr. Arend von Stackelberg	7
0115	Klinikum der Universität München - Campus Großhadern / München	Germany	PD Dr. med. Johanna Tischer	7
0131	UKE Hamburg - Klinik für Stammzelltransplantation / Hamburg	Germany	Prof. Dr. Francis Ayuk	7
0402	Centre Hospital of South-Pest - Saint László Hospital Campus / Budapest	Hungary	Dr. Eszter Sári & Dr. Hardi Apor	5
0401	Szent László Kórház Telephely / Budapest	Hungary	Dr.med. Krisztián Kállay	3
0706	CHU Lille - Institut de Pharmacie / Lille	France	Dr. David Beauvais	3
0702	CHU de Rouen - Centre Pediatric de Transplantation de Cellules Souches Hematopoitiques / Rouen	France	Dr. Nimrod Buchbinder	1
0703	CHRU de Nancy - Onco-hématologie pédiatrique / Nancy	France	Dr. Cecile Pochon	1
0704	CHU Angers / Angers	France	Dr. Corentin Orvain	1
0901	Kinderspital Zürich / Zürich	Switzerland	Prof. Dr. Tayfun Güngör	3
0116	Klinikum Göttingen - Abteilung Hämatologie Onkologie / Göttingen	Germany	Prof. Dr.Gerald Wulf	5
0120	Universitätsklinik Köln / Köln	Germany	Prof. Dr. Udo Holtik	5
0114	Universitätsklinikum Jena - Klinik für Kinder- und Jugendmedizin / Jena	Germany	Prof. Dr. Bernd Gruhn	4
0105	Universitätsklinikum Regensburg / Regensburg	Germany	Dr. Jürgen Föll	3
0106	Universitätsmedizin Greifswald / Greifswald	Germany	Prof. Dr. William Krüger	3
0125	Universitätsklinikum Kiel / Kiel	Germany	Dr. Antonia Vorsterman von Oijen	3
0122	Klinikum Schwabing / München	Germany	Dr. Nadine Anstötz;	2
0130	Universitätsklinikum Münster / Münster	Germany	Prof. Dr. Matthias Stelljes	2
0134	UKSH Lübeck - Klinik für Hämatologie und Onkologie / Lübeck	Germany	Dr. Friederike Wortmann	2
0139	Universitätsklinikum Aachen / Aachen	Germany	Dr. Fabian Beier	2
0117	Universitätsklinikum des Saarlandes / Homburg	Germany	Dr. Konstantinos Christofyllakis	1
0121	Universitätsklinikum Düsseldorf / Düsseldorf	Germany	Dr. Mustafa Kondakci	1
0128	Charité – Hämatologie und Onkologie CBF / Berlin	Germany	Dr. Axel Nogai	1
0129	Charité - Hämatologie, Onkologie und Tumorimmunologie CVK / Berlin	Germany	Dr. Christoph-Philipp Kemna	1
0132	Universitätsklinikum Leipzig / Leipzig	Germany	Dr. Stefanie Petzold-Quinque	1
0133	Medizinische Hochschule Hannover - Pädiatrische Hämatologie und Onkologie / Hannover	Germany	Prof. Dr. Britta Maecker-Kolhoff	1
0135	Universitätsklinikum Regensburg - Klinik und Poliklinik für Innere Medizin III / Regensburg	Germany	Prof. Dr. Daniel Wolff	1
0136	Universitätsklinikum Freiburg / Freiburg	Germany	PD Dr. Brigitte Strahm	1
0137	Universitätskinderklinik Würzburg / Würzburg	Germany	Prof. Dr. Matthias Wölfl	1
0141	Universitätsklinikum Jena - Adults / Jena	Germany	Prof. Dr. Inken Hildendorf	1
0142	Universitätsklinikum Ulm / Ulm	Germany	Prof. Dr. Ansgar Schulz	1
0143	Universitätsklinikum Tübingen - Medizinische Klinik - Innere Medizin II / Tübingen	Germany	Maximilian Reiff	1
